# Mitochondrial complex II and reactive oxygen species in disease and therapy

**DOI:** 10.1080/13510002.2020.1752002

**Published:** 2020-04-14

**Authors:** Katerina Hadrava Vanova, Michal Kraus, Jiri Neuzil, Jakub Rohlena

**Affiliations:** a Institute of Biotechnology of the Czech Academy of Sciences, Prague-West, Czech Republic; b School of Medical Science, Griffith University, Southport, Qld, Australia

**Keywords:** Respiratory complex II, succinate dehydrogenase, reactive oxygen species, OXPHOS, mitochondria, cancer, succinate, tricarboxylic acid cycle

## Abstract

Increasing evidence points to the respiratory Complex II (CII) as a source and modulator of reactive oxygen species (ROS). Both functional loss of CII as well as its pharmacological inhibition can lead to ROS generation in cells, with a relevant impact on the development of pathophysiological conditions, i.e. cancer and neurodegenerative diseases. While the basic framework of CII involvement in ROS production has been defined, the fine details still await clarification. It is important to resolve these aspects to fully understand the role of CII in pathology and to explore its therapeutic potential in cancer and other diseases.

## Introduction

For decades, reactive oxygen species (ROS) have captivated many researchers because of their intractable nature and both beneficial and detrimental roles in cell physiology and pathology. Mitochondria, the site of cellular respiration, are considered the primary site of endogenous ROS production in most cell types. Depending on their concentration, ROS can initiate diverse cellular actions. At physiological levels, they support signaling pathways involved in cell growth and protection, while their high levels lead to cellular damage followed by cell death. The balance between ROS generation and ROS scavenging needs to be tightly regulated [1–3]. Indeed, mitochondrial ROS production has been connected to numerous pathological conditions including neurodegenerative diseases [4], aging [5], oxidative damage during ischemia/reperfusion injury [6], and cancer [7,8].

Initially, respiratory Complex I (NADH:ubiquinone oxidoreductase, CI) and Complex III (ubiquinol:cytochrome c oxidoreductase, CIII) were considered the main sources of mitochondrial ROS, while the contribution of Complex II (succinate dehydrogenase, SDH, CII) was overlooked [9–11]. Identification of mutations in CII resulting in increased ROS production in cancer and neurodegenerative diseases [12–14] and realization that CII plays a crucial role in ROS production also during the reverse electron transfer (RET) through CI [15–18] changed the traditional view. Presently we know that CII contributes significantly to ROS both directly and indirectly (via RET), with important implications in physiology and disease. Mutations in CII are associated with familiar and sporadic forms of cancer, particularly with pheochromocytoma/paraganglioma (PHEO/PGL), gastrointestinal stromal tumors (GIST), and renal cancer, but also with the Leigh syndrome, a neurodegenerative disease. Moreover, CII inhibitors are cardioprotective in ischemia/reperfusion injury (I/R), and can be also applied to cancer therapy [19].

## Complex II structure and function

The research on mitochondrial CII as a source of redox cofactors dates back more than 6 decades [20]. CII is unique in linking the tricarboxylic acid (TCA) cycle and the respiratory chain. CII catalyzes the oxidation of succinate to fumarate, which does not directly contribute to the generation of the proton motive force, but concurrently transfers two electrons derived from this reaction to membrane-bound ubuiquinone. Ubiquinone is thereby reduced to ubiquinol, which fuels CIII and CIV. Interestingly, unlike the other members of the respiratory chain, none of CII subunit is encoded by mitochondrial DNA [21].

**Figure 1.**
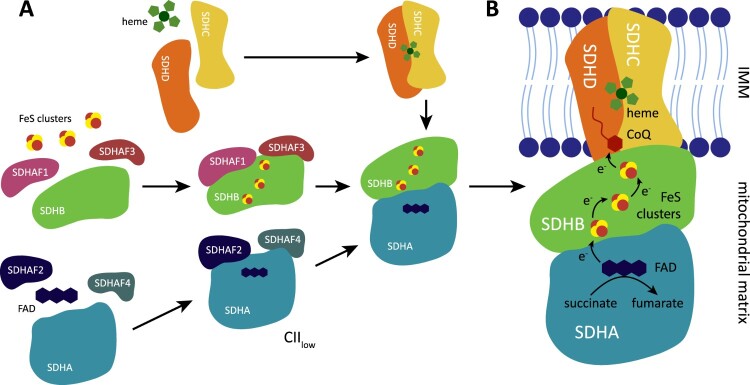
Complex II assembly and its metabolic activity. (A) CII is assembled from four structurally different subunits SDHA-D. SDHA, with covalently bound FAD, requires two assembly factors SDHAF2 and SDHAF4 that assist with SDHA maturation and flavinylation. SDHB contains three iron-sulphur (FeS) clusters, and two additional assembly factors SDHAF1 and SDHAF3 are needed for their insertion. Dimer of SDHA/B is then linked to transmembrane subunits SDHC/D. Under some conditions, an alternative assembly species of CII consisting of SDHA, SDHAF2, and SDHAF4 (described as CII_low_) is stabilized and has an independent biological function. (B) Metabolic activity of the mature CII. CII promotes oxidation of succinate to fumarate. Electrons from succinate are removed by FAD in SDHA and then passed to FeS clusters of SDHB subunit. FeS cofactors transfer the electrons to ubiquinone (CoQ) within the ubiquinone binding (Q) site formed by SDHB, SDHC, and SDHD.

Human CII consists of four subunits, SDHA-D (Figure 1). SDHA is the largest subunit and contains an active site with covalently bound flavine adenine dinucleotide (FAD), which removes electrons from succinate. SDHB carries three linearly aligned iron–sulfur clusters that mediate electron transfer to the ubiquinone molecule located in the ubiquinone-binding (Q) site, jointly formed by SDHB, SDHC, and SDHD. Subunits SDHC and SDHD anchor CII to the mitochondrial inner membrane [22,23]. Four assembly factors participate in CII biogenesis [24–27]. SDHAF2 and SDHAF4 are involved in the maturation of SDHA, and may facilitate covalent flavinylation [24,27,28]. SDHAF1, assisted by SDHAF3, promotes insertion of Fe-S clusters into SDHB [25,26]. Mature SDHA is attached to mature SDHB to be linked to membrane-bound SDHC/SDHD. While the crystal structure of CII was described more than 10 years ago [21,22], additional alternative assembly forms were discovered recently in bacteria [29–31]. One such alternative CII, designated CII_low_ and containing SDHA, SDHAF2, and SDHAF4, was identified also in mammalian cells. CII_low_ is induced in the absence of SDHB or during respiratory dysfunction and may orchestrate cellular adaptations to energy stress [32].

## Complex II and generation of ROS

**Figure 2.**
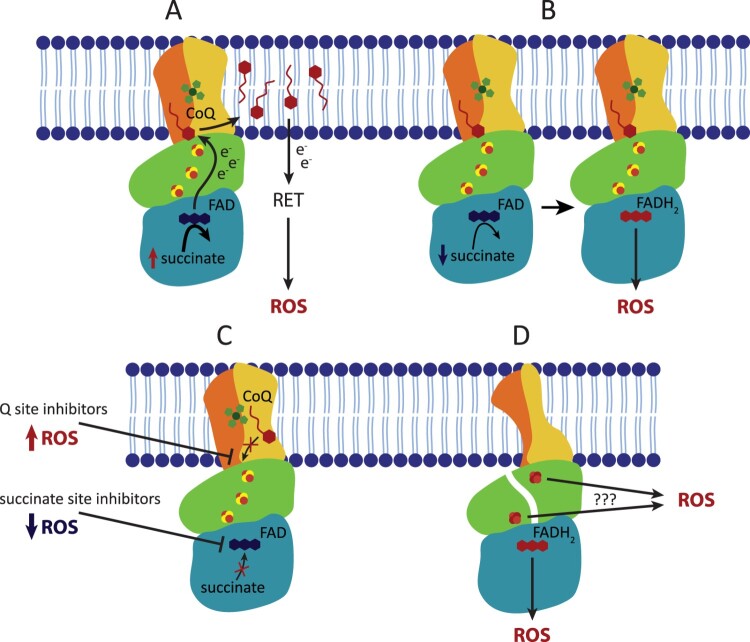
Complex II contributes to ROS production in both physiological and pathophysiological conditions. (A) In the presence of high concentrations of succinate, CII does not produce ROS directly but can contribute to indirect ROS generation via reverse electron transfer (RET) by forcing the electrons onto CI. (B) At lower, physiological succinate concentration, succinate molecule passes the electrons to FAD forming FADH2 which is then able to react with oxygen within the unoccupied succinate binding site, therefore directly forming ROS. (C) The ROS generating ability of reduced FAD is significantly increased when the Q site is blocked by an inhibitor. In contrast, succinate binding site inhibitors block ROS production. (D) Incorrect assembly or damage to CII subunits can induce ROS formation either via reduced FAD or possibly via exposed FeS clusters.

In recent years, it has become apparent that CII has an important role in ROS production. This role is either direct, when ROS are generated at CII, or indirect, when ROS are produced at other sites from electrons supplied by CII (Figure 2). The indirect role was described first, because of the old observation that isolated mitochondria produce large amounts of ROS in the presence of high concentrations (≥5 mM) of succinate, the substrate of CII [15–17,33,34], implicating CII in the process. This is due to RET, when succinate-derived electrons from CII reduce the ubiquinone pool, and electrons are forced backwards from ubiquinone towards CI, where vast quantities of ROS are formed. It was suggested that also CII can produce ROS under these conditions, but this is somewhat controversial and may be tissue-specific [35].

The direct role of CII in ROS production went long unrecognized. This is because the primary ROS producing site in CII, FAD in SDHA, cannot generate ROS when succinate concentration is high (≥5 mM). The mechanism of succinate-mediated inhibition of ROS production at FAD is not entirely clear, but succinate may block access of oxygen to FAD [36]. Respiratory measurements are traditionally performed at 5-10 mM succinate, which masks FAD contribution to ROS production. At 0.5 mM succinate, a concentration similar to normal intracellular succinate levels, the contribution of CII’s FAD to ROS generation can be substantial when electron transport through CII is blocked at the Q site or further downstream (at CIII, for example), suggesting that ROS is produced when FAD is reduced, but the active site is not occupied [36,37]. Under specific conditions, ROS generation was also observed at the Q site [38], however, this is likely infrequent in mammalian CII.

Inhibitors of CII show ambivalent effect on mitochondrial ROS production depending on substrate supply, membrane potential and overall metabolic activity of the cell as well as intracellular succinate concentration [39,40]. Specific inhibitors of CII bind either to the succinate-binding site, i.e. oxaloacetate and malonate (reviewed in [14]), or to the Q site, i.e. thenoyltrifluoroacetone (TTFA) [22], atpenin [41], α−tocopheryl succinate [42], or mitochondrially targeted vitamin E succinate (MitoVES) [43–45]. Generally, succinate-binding site inhibitors suppress ROS production from CII as they block FAD, while Q site inhibitors stimulated ROS generation as they reduce FAD by blocking electron transfer to ubiquinone. However, in intact cells only intermediate affinity (TTFA, MitoVES) Q site inhibitors induce ROS (and cell death), while high-affinity Q site inhibitors such as atpenin do not induce ROS [45]. The explanation is that the plasma membrane is impermeable for succinate, and succinate rapidly accumulates when high-affinity Q site inhibitors are employed, canceling ROS production from FAD. This is perhaps the case for atpenin A5, which immediately blocks all CII molecules in a cell, so that succinate cannot be consumed. Lower affinity Q site inhibitors do not occupy all CII molecules at the same time, which keeps succinate down, allowing ROS production at FAD of those CII molecules that have the Q site blocked. Indeed, atpenin treatment [46], unlike TTFA and MitoVES, do not induce ROS-mediated cell death [45], and atpenin is quite well tolerated by cultured cells. Finally, both the succinate-binding site and the Q site inhibitors suppress ROS production under high succinate concentrations during RET [47] as they all prevent the transfer of electrons from succinate via CII to the ubiquinone pool.

## The paradox of ROS production from CII

It has been shown that functional loss of CII can lead to succinate accumulation and ROS generation in cells [19]. Guzy et al. found that pharmacological inhibition of CII or silencing of *SDHB* can lead to ROS production and ROS-dependent stabilization of hypoxia-inducible factor-α [48], while others ascribed this effect to the accumulation of succinate [49]. Similarly, CII dysfunction, increased ROS formation, and mtDNA mutability were observed in a yeast model with mutated *SDHB* [50]. Mutations in the *SDHC* subunit of CII in fibroblasts from a transgenic mouse enhance ROS generation due to dysfunction of mitochondrial respiration [51]. Similarly, downregulation of the expression of the SDHC subunit in hepatocellular carcinoma was linked to increased cancer cell growth and metastasis due to elevated ROS production with subsequent nuclear factor-κB signaling [52]. A study using hamster fibroblasts revealed that mutation in *SDHD* resulted in elevated ROS production [53]. A similar effect on the production of ROS and instability of DNA was observed in yeast mutant of *SDH* [54].

These observations are puzzling given recent strong evidence for FAD in SDHA being the principal site of ROS production in the mature mammalian CII, coming both from isolated mitochondria and from intact cells [36,37,45]. We face the following paradox. Mutations and/or manipulations that interfere with CII and therefore favor reduced FAD will also increase intracellular succinate to concentration over 5 mM which is incompatible with ROS production from FAD in mammalian CII. Indeed, PHEO/PGL-associated mutations in the *SDHC* subunit that stimulate ROS at low (0.5 mM) succinate levels in isolated mitochondria often do not stimulate ROS in intact cells [45]. There are several relevant aspects that should be considered when thinking about CII-derived ROS in pathology. When wild-type CII alleles are present (heterozygous mutations, incomplete knockdown), these will control succinate levels to some degree to allow ROS production at FAD by mutated CII. Indeed, inherited PHEO/PGL-associated germline mutations are heterozygous during tumor development. Yeast results could perhaps be explained by a different behavior of mammalian/*Escherichia coli* CII compared to *Saccharomyces cerevisiae* CII with respect to ROS production. While the amount of ROS produced at different succinate concentrations follows the typical bell-shaped curve for human and *E. coli* CII (with a maximum at about 0.5 mM succinate, corresponding to a typical concentration in normal cells) [36,47,55], this is not the case for *S. cerevisiae* CII. In yeast, ROS production at CII is succinate-insensitive and the likely source is the Q site [56,57]. For this reason, yeast CII may not be the optimal model to study ROS-related aspects of CII-dependent tumorigenesis.

Improperly assembled CII, for example incorrect insertion of FeS clusters into SDHB, can result in increased ROS [26]. Yet, Maklashina et al. showed that free *E. coli* SDHA flavoproteins have only minor catalytic activity and generate little or no ROS. Their results suggest that the iron–sulfur protein SDHB in CII is necessary for robust catalytic activity and ROS generation by incomplete CII [58]. This could explain how CII could produce ROS to amplify the apoptotic response. In this scenario, SDHA/SDHB subcomplex disengages from the membrane-bound SDHC/SDHD, and superoxide is formed [59]. The precise site of superoxide generation was not identified, but it could possibly originate from the exposed FeS clusters of SDHB that would be insensitive to succinate inhibition. This raises the possibility that CII mutations, which can alter CII conformation (particularly in SDHB), could allow ROS production even in the presence of accumulated intracellular succinate, circumventing the FAD paradox.

## CII in disease

Isolated defects of CII are relatively rare, accounting for approximately 2% of all respiratory chain deficiency diagnoses [60]. Still, accumulating evidence links *SDHx* mutations to pathology of the nervous system and the brain. Deficiency of CII is recognized to cause encephalomyopathy in childhood and optic atrophy in adulthood [61]. Jain-Ghai et al. reviewed 37 clinical cases of CII deficiency, concluding that neurological findings, abnormal brain imaging, and developmental delay were the most common manifestation of CII defects, regardless of the large variation in the phenotype [62]. Chronic administration of 3-nitropropionic acid (3-NPA), an irreversible inhibitor of succinate dehydrogenase, replicates the neuropathologic and clinical features of Huntington disease (HD) in nonhuman primates [63]. Later it was shown that patients with HD have severe defects of CII in caudate nucleus [64], which can mediate striatal cell death and neurodegeneration mimicking the development of HD [65]. On the other hand, Naseri et al. recently measured an elevated SDH activity in HD patient lymphoblasts [66], pointing to a possible compartment-specific CII regulation.

One of the rare cases of documented autosomal inheritance of SDHA subunit defect was linked to bilateral optic atrophy, ocular movement disorder, progressive polyneuropathy, psychiatric involvement, and cardiomyopathy [60]. Mutations in *SDHA*, *SDHB* and *SDHAF1* were reported in leukodystrophy [67], Leigh syndrome and cardiomyopathy [23,68–70], and infantile leukoencephalopathy [25]. Recently, a case of encephalomyopathy has been connected to a recessive germline mutation in *SDHD* subunit [71]. Moreover, assembly factor SDHAF4 was implicated in neuroprotection, possibly decreasing ROS generated by the free SDHA subunit [27]. Hence, ROS production via CII may play a role in neurodegenerative processes.

SDHx defects show a strong association with tumorigenesis, and *SDHx* genes are considered tumor suppressors. Germline mutations in subunits *SDHA-D*, as well as assembly factor *SDHAF2*, were recognized to cause familial PHEO/PGL [13,23,72]. Further, SDH dysregulation is linked to GIST oncogenesis [23,73] and renal carcinoma [74,75], but less frequently. In addition, the familiar *SDHx* defects are connected to PTEN mutation-negative Cowden syndrome, associated with breast, thyroid, and endometrial neoplasias [76].

Unlike in neurological disorders and cancer, in other pathologies, the direct genetic link to CII has not been established. However, evidence is emerging for the role of mitochondrial ROS in obesity [77–79], insulin resistance/diabetes [79,80], cardiovascular diseases [81], and non-alcoholic fatty liver disease [79,82,83]. With regard to CII/ROS, skeletal muscle biopsies from patients with obesity and diabetes showed changes in CII activity [78,84,85]. Also visceral adipose tissue in obese patients exhibits decreased CII activity compared to subcutaneous adipose tissue which can be restored *in vitro* by addition of the mitochondria-specific oxidant scavenger mito-TEMPO [77]. Chemical inhibition of CI and CII by amiodarone followed by increased ROS production may result in steatohepatitis [86]. Moreover, Fazakerley et al. have suggested that loss of mitochondrial CoQ can drive adipocyte insulin resistance most likely via CII-dependent mitochondrial ROS production [87]. Altogether, CII should be considered when searching for novel therapeutic approaches in metabolic disorders.

## Targeting CII/ROS as a therapeutic approach

Mitochondrial malfunction and increased ROS production are relevant in aging, neurodegenerative diseases, obesity, diabetes, and cancer [88,89]. ROS can be countered by antioxidants, but the therapeutic application of antioxidants has yielded disappointing results, possibly because only a small fraction of these compounds are taken up by mitochondria [88]. Hence, mitochondrial targeting was employed to accumulate antioxidants within mitochondria [89]. One of the best characterized mitochondria-targeted antioxidants is mitochondrially targeted coenzyme Q (MitoQ) containing the triphenylphosphonium (TPP^+^) moiety (reviewed in [89,90]). In mitochondria, the reduced form of MitoQ is oxidized, followed by its rapid re-reduction at CII, which was documented to act as a protective mechanism in different cell models of mitochondrial oxidative stress [91] and neuroprotection [92]. Furthermore, MitoQ was studied in metabolic syndrome and proved to be effective against hypercholesterolemia, hypertriglyceridemia, mtDNA oxidative damage, hyperglycemia, and hepatic steatosis (reviewed in [83]).

Mitochondrial ROS production is involved in I/R injury, and CII inhibitors exert protective effects in different I/R models by suppressing RET [93–96]. Mitochondria-targeted tanshinone IIA, a new CII inhibitor, was developed and showed to be protective in I/R oxidative injury [97]. A similar effect was shown for the ferulic acid derivative hmy-paa (3-(4-hydroxy-3-methoxyphenyl)-N-(1H-pyrazol-3-yl)acrylamide) [98]. This is because during the ischemic phase of I/R accumulated succinate is quickly oxidized upon oxygen availability, resulting in massive RET and ROS generation at CI. CII inhibitors, such as malonate, that prevent electron transfer through CII to the ubiquonine pool, therefore, prevent RET and ROS production, are protective during I/R or cold ischemia [99,100]. However, it has also been proposed that CII-dependent reserved respiratory capacity affords cardioprotection during cardiomyocyte recovery from hypoxia [101].

Mills et al. showed that CII-induced ROS production by RET is involved in LPS-stimulated macrophage activity. Succinate-dependent ROS generation was observed, resulting in pro-inflammatory responses, while inhibition of CII by malonate promoted an anti-inflammatory outcome [102]. Interventions at CII can thus regulate inflammation which is associated with numerous metabolic and cardiovascular disorders [103]. Cardioprotective effects of diazoxide was linked directly to inhibition of SDH [104]. In addition, inhibition of CII with 3-NPA reduced glucose-stimulated insulin secretion and ROS production, thereby offering new directions in treatment of cell damage in diabetes [105]. Conversely, while most therapies are focused on inhibiting CII and ROS production, stimulation of SDH activity by succinate administration drives production of ROS and thermogenic respiration in brown adipose tissue, which may stimulate protection against diet-induced obesity and improve glucose tolerance [106]. These findings suggest that targeting CII and CII-driven ROS production may broaden the potential treatment of metabolic disorders.

It has been proposed that CII may function as a general sensor for apoptosis [59,107], making CII a regulator of cell death [108]. Indeed, blockade of the Q site of CII can induce apoptosis by stimulating ROS production from FAD. The amplitude of cell death is directly proportional to the amount of CII-produced ROS [45]. Thus, CII can be targeted for cancer therapy, and efficient experimental anti-cancer agents directed at the Q site have been developed [42–44,109]. The list of potential chemicals to manipulate CII and CII-dependent ROS has been recently updated, including α-TOS, mitoVES, 3-bromopyruvate, malonate, 3-NPA, TTFA, atpenins, lonidamine, and DT-010 as possible candidates for cancer therapy [110]. In addition to the direct effect on cancer cells, some agents also reduce tumor angiogenesis [111,112]. Furthermore, non-toxic doses of the Q site inhibitor TTFA sensitize cancer cells to cell death regulated by other drugs [113], suggesting that CII has potential in combinational cancer therapy. This is in line with CII being an important player in cell death induction. Additionally, it has recently been shown that tumors carrying *SDHB* mutations produce more ROS and accumulate iron, and disruption of redox hemostasis by ascorbic acid to induce cell death seems to be a promising tool for the treatment of *SDHB*-mutated PGL/PHEO [114].

## Conclusions

Accumulating evidence suggests that CII is an important and underestimated source and modulator of ROS in physiological and pathophysiological conditions that can be manipulated to both induce and suppress cell death, depending on the scenario. Since literature on the therapeutic application of CII modulation in cancer, neurodegeneration, and other pathologies is still fractional, a better understanding of the basic mechanisms of ROS regulation by CII in disease may lead to new therapeutic approaches.
